# Mitochondrial Network Fragmentation Leads to Dysfunction of Macrophages During *Echinococcus multilocularis* Protoscoleces Infection

**DOI:** 10.3390/pathogens14111097

**Published:** 2025-10-28

**Authors:** Zihan Yang, Yaogang Zhang, Tao Zhang, Jing Hou, Meiyuan Tian, Dengliang Huang, Yuan Jiang, Li Sun, Panlong Wei, Yanyan Ma

**Affiliations:** 1 Research Center for High Altitude Medicine, Qinghai University, Xining 810000, China; 2 Key Laboratory of High-Altitude Medicine (Ministry of Education), Qinghai University, Xining 810000, China; 3 Key Laboratory of Application and Foundation for High Altitude Medicine Research in Qinghai Province (Qinghai-Utah Joint Research Key Lab for High Altitude Medicine), Qinghai University, Xining 810000, China; 4 Department of Neurology, Qinghai University Affiliated Hospital, Xining 810000, China; 5 Central Laboratory of Qinghai University Affiliated Hospital, Qinghai University Affiliated Hospital, Xining 810000, China; 6 Qinghai Province Research Key Laboratory of Echinococcosis, Qinghai University Affiliated Hospital, Xining 810000, China; 7 Department of Rehabilitation Medicine, Qinghai University Affiliated Hospital, Xining 810000, China; 8 Department of Pediatrics, Qinghai University Affiliated Hospital, Xining 810000, China

**Keywords:** *Echinococcus multilocularis*, protoscoleces, mitochondrial fragmentation, macrophage phagocytosis

## Abstract

Alveolar echinococcosis (AE) is a fatal foodborne parasitic disease caused by the larvae of *Echinococcus multilocularis*. The disease primarily affects the liver. Previous studies have found that Kupffer cells have an immune protective effect, but in the late stages of AE, they are associated with parasite immune escape. The present study analyzed the effects of *Echinococcus multilocularis* protoscoleces (PSCs) infection on the mitochondrial morphology and function of macrophages, as well as their phagocytic function and apoptosis. Infection with PSCs has been shown to result in the fragmentation of the macrophage mitochondrial network, the impairment of mitochondrial membrane potential, the elevation of mitochondrial reactive oxygen species, and the reduction in mitochondrial DNA copy number. This cascade of events, consequent to the infection, has been demonstrated to promote the apoptosis of macrophages and impair their phagocytic function. Inhibiting mitochondrial fission during PSCs infection has been shown to mitigate mitochondrial dysfunction, suppress macrophage apoptosis, and enhance macrophage phagocytic function. This discovery provides insights into improving macrophage function during the progression of AE.

## 1. Introduction

Alveolar echinococcosis (AE) is a foodborne parasitic disease caused by the larvae of *Echinococcus multilocularis* (*E. multilocularis*). The disease is predominantly prevalent in the Northern Hemisphere, with an estimated median of 10,489 new AE cases annually, 92% of which occur in China [1]. The median estimate of the total number of disability-adjusted life years (DALYs) worldwide each year is 666,433 [2]. Humans are intermediate hosts of *E. multilocularis*. Infection is often caused by accidentally ingesting food or water contaminated with parasite eggs. The liver is the organ most frequently affected [2]. In the absence of treatment or effective therapy, the mortality rate among patients with confirmed AE can exceed 90% within 10–15 years after diagnosis [3].

Previous studies have found that Kupffer cells, a type of resident macrophage in the liver, have an immune protective effect against *E. multilocularis* [4]. Macrophages accumulate at AE lesions, where they initially exert pro-inflammatory effects upon *E. multilocularis* infection and adhere to the surface of *E. multilocularis* protoscoleces (PSCs), disrupting their structural integrity and ultimately inducing PSCs death [5]. Conversely, in the late stages of *E. multilocularis* infection, macrophages adopt a predominantly anti-inflammatory profile, with diminished capacity to kill PSCs, thereby sustaining immune tolerance and fostering progressive liver fibrosis [6,7,8]. This phenotypic switch may be linked to mitochondrial dynamics. Susser et al. reported that in bone marrow-derived macrophages (BMDMs), mitochondrial fragmentation enhances arginase-1 expression via increased histone lactylation, thereby promoting inflammatory resolution responses [9]. However, whether an analogous mitochondrial-dependent switch operates in macrophages during AE remains to be determined.

Mitochondria serve as a molecular platform for integrating innate immune signals, participating in immune responses by activating mitochondrial antiviral-signaling protein (MAVS) and producing mitochondrial reactive oxygen species (mtROS) and cytokines [10]. Mitochondrial dynamics have been implicated in macrophage polarization [11], activation of the NOD-like receptor family pyrin domain-containing 3 (NLRP3) inflammasome in macrophages [12], and the phagocytic clearance of apoptotic cells by macrophages [13].

Pathogens can also promote their replication or survival by altering the morphology of host macrophage mitochondria. *Legionella pneumophila* injects effector proteins into macrophages via the type IV secretion system. The effector protein MitF induces dynamin-related protein 1 (DRP1)-dependent mitochondrial fragmentation, thereby promoting the Warburg effect, which converts mitochondrial oxidative phosphorylation in macrophages to glycolysis and facilitates the replication of *Legionella pneumophila* within macrophages [14]. During *Listeria monocytogenes* infection, macrophages can be activated and defend against infection [15]. *Listeria monocytogenes* transiently fragments mitochondria of epithelial cells by up-regulating Mic10—an inner mitochondrial membrane protein involved in cristae formation—via listeriolysin O, thereby fostering bacterial proliferation [16,17]. The main target cells of dengue virus (DENV) infection are myeloid cells (monocytes, macrophages, and dendritic cells) [18]. DENV has been observed to suppress mitochondrial fission in Huh7 cells by down-regulating DRP1 expression, and inducing fission has been shown to reduce viral replication [19]. DENV has also been observed to cleave mitofusins via the viral protease NS2B3 in A549 cells, thereby disrupting Mitofusin 1 (MFN1)-mediated mitochondrial fusion, dampening IFNβ expression, and consequently facilitating viral replication [20,21]. *Leishmania donovani* infection reduces Mitofusin 2 (MFN2), leading to increased mitochondrial fission in macrophages, which limits the turnover of microRNAs and inhibits the production of pro-inflammatory cytokines, thereby promoting the survival of the parasite within the cell [22]. *Chlamydia trachomatis*-induced upregulation of miR-30c reduces p53 expression, thereby decreasing the expression and accumulation of functional DRP1 on the mitochondrial surface. This leads to mitochondrial elongation and excessive fusion, ensuring ATP production in cells and facilitating the survival of *Chlamydia trachomatis* [23]. How the aforementioned pathogens manipulate host–cell mitochondrial dynamics to favor their survival or replication is summarized in Table 1.

*E. multilocularis* infection induces an augmentation of mitochondrial fission in the murine hepatocyte cell line AML12 [24]. However, the question of whether *E. multilocularis* infection modulates mitochondrial dynamics in macrophages to promote parasite survival in vivo remains to be resolved. We hypothesize that *E. multilocularis* may also impair macrophage function by altering host macrophage mitochondrial dynamics, thereby facilitating parasite persistence.

This study observed the effects of *E. multilocularis* infection on macrophage function and mitochondrial morphology and function. The quinazolinone derivative termed Mdivi-1 could inhibit DRP1 GTPase activity to attenuate mitochondrial fission [25]. In vitro studies have demonstrated the efficacy of Mdivi-1 in suppressing mitochondrial fission in various macrophages, including Raw264.7, THP-1, and peritoneal macrophages [26,27,28]. Utilizing Mdivi-1 in Raw264.7 cells, we examined whether inhibition of mitochondrial fission affects macrophage mitochondrial and cellular functions during *E. multilocularis* infection, aiming to explore the pathogenesis of AE from the perspective of mitochondrial dynamics.

## 2. Materials and Methods

### 2.1. Cell Culture

The murine macrophage cell line Raw264.7 was derived from the Cell Bank of the Chinese Academy of Sciences (Shanghai, China). Raw264.7 cells were cultured in Dulbecco’s Modified Eagle Medium (DMEM) (Gibco, 12800017, New York, NY, USA) containing 10% certified fetal bovine serum (VivaCell, C04001-500, Shanghai, China) in a 37 °C incubator (Thermo, 3131, Scoresby, VIC, Australia) containing 5% carbon dioxide.

### 2.2. Isolation and Culture of PSCs

PSCs originated from wild-caught *Microtus arvalis* infected with AE. The obtained PSCs were injected into *Mongolian gerbils* (Hangzhou, China) via intraperitoneal injection. Three months later, the gerbils were anesthetized with isoflurane (RWD Life Science, R510-22-10, Shenzhen, China) and euthanized. The AE lesions in the gerbils were separated, chopped, and ground in sterile culture dishes, then filtered through 80 μm and 300 μm aseptic filters (Solarbio, Beijing, China). The isolated PSCs were cultured in DMEM containing 20% fetal bovine serum. Their viability was assessed by 0.1% trypan blue staining, and PSCs with a survival rate greater than 90% were used to infect Raw264.7 for the experiment. The study protocol was approved by the Ethics Committee of the Qinghai University Affiliated Hospital (Approval No: P-SL-2023143).

### 2.3. E. multilocularis Infection

According to our previous protocol [7], PSCs were added after Raw264.7 cells have adhered to the culture dish (Raw264.7: PSCs = 500:1). PSCs were gently washed with phosphate-buffered saline (PBS) at 24 h, 48 h, and 72 h after infection, and the cells were collected for subsequent experiments. For the mitochondrial fission inhibition group, the mitochondrial fission inhibitor Mdivi-1 was added to Raw264.7 at a concentration of 20 μM during the last 6 h of the 72 h infection period of PSCs.

In the AE mouse model, 6–8-week-old C57BL/six mice (purchased from Cyagen Biosciences, Santa Clara, CA, USA) were used. Under isoflurane anesthesia, 1500 PSCs were injected into the liver in situ under the guidance of a Mindray Veterinary Diagnostic Ultrasound System (Vetus 5, Shenzhen, China). Three months later, the mice were euthanized, and liver tissue surrounding the lesions was collected for hematoxylin and eosin (HE) staining and immunofluorescence. The study complied with the ethical approval of the Qinghai University Affiliated Hospital (P-SL-2023143).

### 2.4. Human Subjects

The liver tissue pathology sections were obtained from AE patients who underwent surgical treatment at Qinghai University Affiliated Hospital. The study was conducted in accordance with the Declaration of Helsinki, and the protocol was approved by the Ethics Committee of the Qinghai University Affiliated Hospital (P-SL-2023143), with all patients having given informed consent.

### 2.5. Immunofluorescence Staining

Lesion liver tissue from AE patients and AE mice resected during surgery was cut into 5 μm thick frozen sections using a cryostat microtome. Dry the frozen sections at room temperature for 10 min, wash with PBS, and incubate with endogenous peroxidase blocking solution (Boster, AR1108, Pleasanton, CA, USA) at room temperature for 10 min. After washing with PBS, use 5% goat serum to block at room temperature for 1 h. Next, use CD68 (1:200, Novus, NB100-683, Minneapolis, MN, USA) to block overnight at 4 °C. The next day, incubate the secondary antibody (Goat anti-Mouse IgG H&L, 1:1000, Abcam, ab150115, Waltham, MA, USA) at room temperature for 1 h. Finally, cell nuclei were stained with DAPI staining solution (Abcam, ab104139, Waltham, MA, USA) and sealed with anti-fluorescence quenching agent. Observations and photographs were taken using a TG TissueFAXS (TissueGnostics, Vienna, Austria) fluorescence microscope.

### 2.6. Mitochondrial DNA (mtDNA) Copy Number

Extract DNA from cells and dilute to 5 ng/μL for subsequent qPCR analysis of nuclear DNA and mtDNA content using a Roche Light Cycler 480 II (Roche, Rotkreuz, Switzerland)in the presence of a Light Cycler^®^ 480 SYBR Green I Master (Roche, Rotkreuz, Switzerland 04887352001). The amplification program was as follows: pre-denaturation at 95 °C for 10 min, followed by 45 cycles of 10 s at 94 °C and 34 s at 60 °C. mtDNA content was determined by normalizing mtDNA abundance (16S rRNA) to nuclear DNA (beta-2-microglobulin, B2M) abundance. The primers used for qPCR were listed as follows [29]: mtDNA 16S rRNA-F (5′-CTAGAAACCCCGAAACCAAA-3′), mtDNA 16S rRNA-R (5′-CCAGCTATCACCAAGCTCGT-3′), B2M (beta-2-microglobulin)-F (5′-ATGGGAAGCCGAACATACTG-3′), and B2M (beta-2-microglobulin)-R (5′-CAGTCTCAGTGGGGGTGAAT-3′).

### 2.7. Macrophage Apoptosis

To assess the impact of PSCs infection and the inhibition of mitochondrial fission during PSCs infection on the viability of Raw264.7 cells, Annexin V-FITC and PI staining were performed using the Apoptosis Detection Kit (BD Pharmingen™ FITC Annexin V Apoptosis Detection Kit I, 556547, San Diego, CA, USA) according to the manufacturer’s instructions. After incubation at room temperature for 15 min, apoptotic cells were detected using flow cytometry. Cell viability was expressed as a percentage and categorized into the following groups: viable cells (FITC Annexin V- and PI-negative), early apoptosis (FITC Annexin V-positive and PI-negative), and late apoptosis and necrosis (FITC Annexin V- and PI positive). The percentage of FITC Annexin V-positive cells was statistically analyzed and compared across groups.

### 2.8. Transmission Electron Microscopy (TEM)

Raw264.7 cells infected with PSCs for 0–72 h were fixed at room temperature for 24 h using 2.5% glutaraldehyde in phosphate buffer, then fixed at 4 °C for 12 h. After washing with 0.1 M phosphate buffer, the cells were fixed with 1% osmium acid for 2 h. After rinsing again with 0.1 M phosphate buffer, the sample was sequentially immersed in the following liquids for dehydration, with each sample being soaked for 15 min: 50% alcohol, 75% alcohol, 80% alcohol, 95% alcohol, 100% alcohol I, 100% alcohol II, 100% acetone I, and 100% acetone II. Immerse the sample in infiltration A (acetone: SPI 812 embedding agent = 2:1) at room temperature for 2 h, then immerse it in infiltration B (acetone: SPI 812 embedding agent = 1:2) at room temperature overnight. After two 2 h infiltrations with SPI 812 embedding agent, the samples were put into embedding molds, and additional embedding agent was added. The samples were then hardened by baking them in an oven at 37 °C and 60 °C for 12 h. Use a Leica UC7 ultramicrotome to cut the samples into 60–80 nm slices. Use a copper mesh to scoop out the slices, then immerse them in a 2% uranyl acetate staining solution for 30 min. Next, rinse the slices with deionized water and immerse them in a lead citrate staining solution for 15 min to complete the lead–uranium double staining. Images were acquired with a JEM-1400 Flash transmission electron microscope, and mitochondrial length was quantified using ImageJ (Version 1.54f).

### 2.9. Fluorescence Microscopy Imaging and Analysis

Inoculate Raw264.7 into 6-well TC-treated plates containing cell-climbing slices. After the cells have adhered, add PSCs (at the same ratio as before) for infection. In the Mdivi-1 treatment group, add Mdivi-1 during the last 6 h of infection and achieve a final concentration of 20 μM. Mitochondria were stained with 100 nM MitoTracker Deep Red (Invitrogen, M22426, Eugene, OR, USA; excitation, 644 nm; emission, 665 nm) for 30 min at 37 °C, and nuclei were counterstained with Hoechst 33258 (Solarbio, IH0060, Beijing, China; excitation, 346 nm; emission, 460 nm) for 20 min at 37 °C. Following fixation in 4% paraformaldehyde at 37 °C for 10 min, slices were sealed with anti-fluorescence quenching agent (BOSTER, AR1109, Pleasanton, CA, USA) and imaged on a Nikon N-SIM+N-S super-resolution confocal microscope. Mitochondrial aspect ratio and circularity were quantified in ImageJ following the protocol of Chaudhry et al. [30].

### 2.10. Mitochondrial Membrane Potential

Mitochondrial membrane potential of Raw264.7 was assessed using the Mitochondrial Membrane Potential Assay Kit with JC-1 (Solarbio, M8650, Beijing, China) according to the manufacturer’s protocol. The chemical uncoupler carbonyl cyanide 3-chlorophenylhydrazone (CCCP) was employed as a positive control. JC-1 exhibits potential-dependent accumulation in mitochondria, indicated by a fluorescence emission shift from green (529 nm) to red (590 nm). An increased proportion of cells containing green fluorescent J-monomers indicates dissipation of mitochondrial membrane potential.

### 2.11. Mitochondrial Derived Reactive Oxygen Species (mtROS)

MitoSOX™ Red mitochondrial superoxide indicator (Invitrogen, M36008) was used strictly according to the manufacturer’s instructions. mtROS was quantified by flow cytometry at an emission wavelength of 605 nm, and the proportion of mtROS-positive cells was statistically analyzed.

### 2.12. Macrophage Phagocytosis

Gently wash away the PSCs with PBS, leaving the Raw264.7 cells adhering to the culture dish. Raw264.7 were then infected with enhanced green fluorescent protein (EGFP)-expressing *Escherichia coli* (*E. coli*) (Miaoling biology, P2162, Wuhan, China) at a multiplicity of infection (MOI) of 10:1 (EGFP-*E. coli*/cell) at 37 °C for 1 h. Wash away extracellular *E. coli* with PBS. Collect Raw264.7 and quantify the phagocytic function of macrophages by measuring the percentage of EGFP-positive cells using flow cytometry.

### 2.13. PSCs Survival Rate

After treating PSCs with 20 μM Mdivi-1 for 6 h, add propidium iodide (PI) (BD Pharmingen™ FITC Annexin V Apoptosis Detection Kit I, 556547) and incubate at 37 °C for 15 min to stain dead PSCs. Images were taken by a Biotek Cytation5 (New York, NY, USA) for statistical analysis of the survival rate of PSCs.

### 2.14. Statistical Analysis

Comparisons between two groups were undertaken using an unpaired *t*-test (two-tailed). Comparisons between three or more groups were analyzed by one-way ANOVA. Pairwise comparisons between multiple groups were performed using Tukey’s multiple comparisons test. Values of *p* < 0.05 were considered statistically significant (* *p* < 0.05, ** *p* < 0.01, *** *p* < 0.001). The calculation of these parameters were carried out using GraphPad Prism version 8.0 software.

## 3. Results

### 3.1. PSCs Infection Causes Macrophage Dysfunction

**Figure 1:**
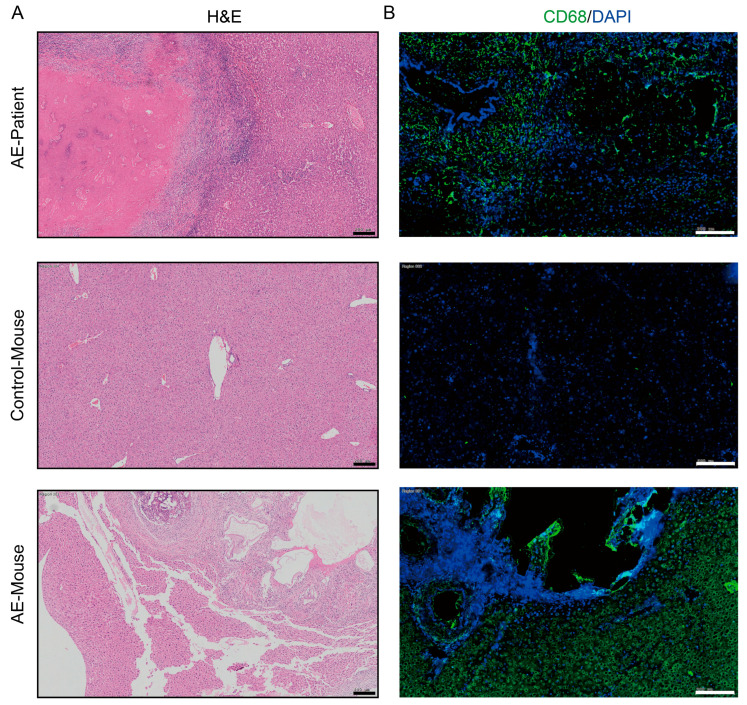
Macrophages accumulate around liver lesions of AE patients (*n* = 8) and AE model mice (*n* = 8). (**A**) HE staining of liver histopathological sections from AE patients and AE model mice (scale bar, 200 μm). (**B**) Immunofluorescence staining for the pan-macrophage marker CD68 in liver tissue samples from patient and mice with AE (scale bar, 200 μm).

**Figure 2:**
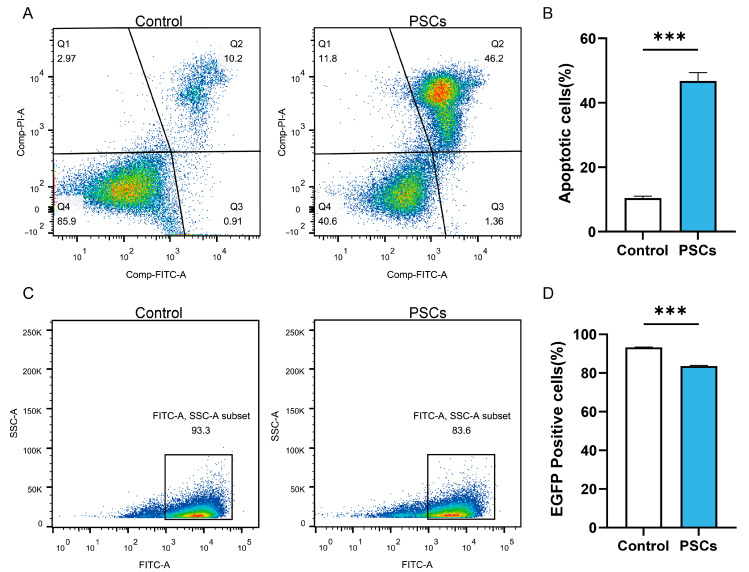
PSCs infection impairs Raw264.7 function. (**A**) The apoptosis of Raw264.7 infected with PSCs for 72 h was detected by flow cytometry. (**B**) Statistics of the Raw264.7 apoptosis rate of each group in (**A**). (**C**) Following a 72 h infection period with PSCs, the percentage of Raw264.7 cells that had phagocytosed EGFP-expressing *E. coli* was detected by means of flow cytometry. (**D**) Proportions of EGFP-positive Raw264.7 cells in each group presented in (**C**) were quantified. Data were collected from three independent biological replicates, each comprising three technical replicates. Statistical analysis was performed using unpaired *t*-test (two-tailed), *** *p* < 0.001.

In AE patients and AE mouse models, macrophages can be observed to accumulate around liver lesions (Figure 1). To clarify the effect of PSCs infection on macrophage function, we infected RAW264.7 murine macrophages with PSCs for 72 h and assessed the apoptosis rate of Raw264.7 and the ratio of Raw264.7 phagocytosing EGFP-labeled *E. coli* via flow cytometry. The results revealed that PSCs infection led to increased macrophage apoptosis (Figure 2A,B) and impaired phagocytic function (Figure 2C,D).

### 3.2. PSCs Infection Impairs Mitochondrial Function of Macrophages

**Figure 3:**
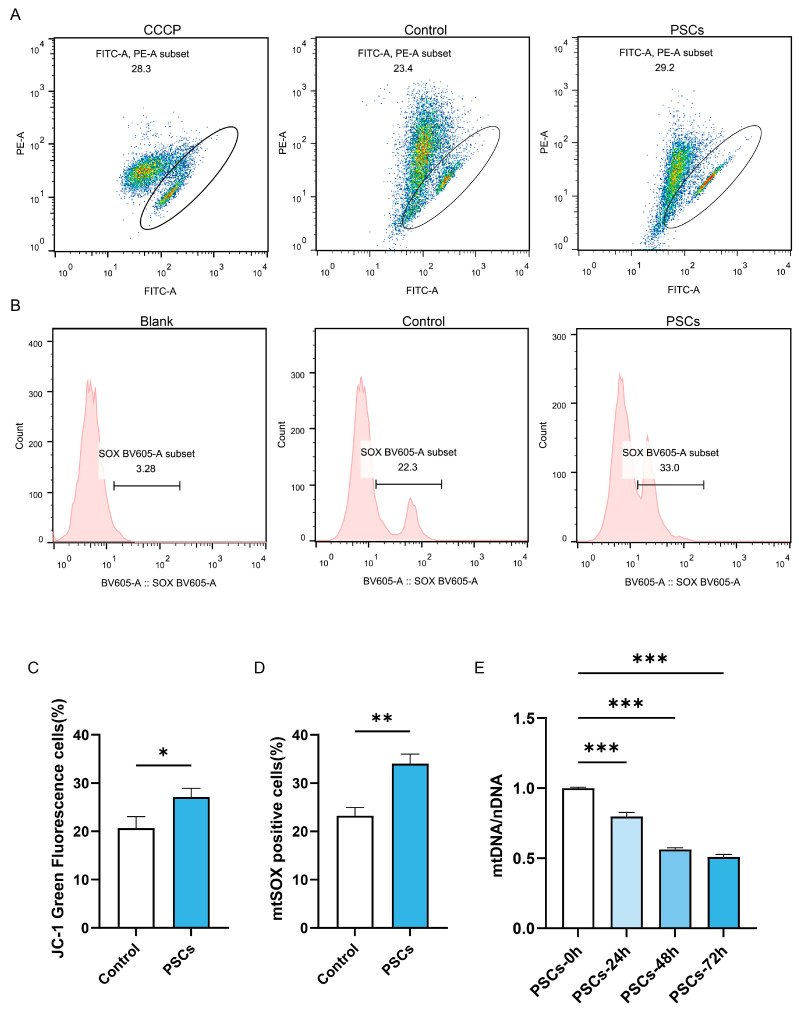
PSCs infection impairs Raw264.7 mitochondrial function. (**A**) The proportion of Raw264.7 cells exhibiting impaired mitochondrial membrane potential after 72 h of PSC infection was assessed by JC-1 staining and flow cytometry. (**B**) The proportion of mtROS-positive Raw264.7 cells had been quantified by flow cytometry after 72 h of PSCs infection. (**C**) Statistics on the proportion of Raw264.7 cells with damaged mitochondrial membrane potential in (**A**). Data were collected from three independent biological replicates, each comprising three technical replicates. Statistical analysis was performed using unpaired *t*-test (two-tailed), * *p* < 0.05. (**D**) Statistics on the proportion of mtROS-positive Raw264.7 cells in each group in (**B**). Data were collected from three independent biological replicates, each comprising three technical replicates. Statistical analysis was performed using unpaired *t*-test (two-tailed), ** *p* < 0.01. (**E**) qPCR was utilized to detect the mtDNA copy number of Raw264.7 infected with PSCs for 0–72 h, normalized to the amount of nuclear DNA. Data were collected from three independent biological replicates, each comprising three technical replicates. Statistical analysis was performed using one-way ANOVA, *** *p* < 0.001.

Since mitochondria serve as the energy factory and immune platform of cells and can also release cytochrome C to promote apoptosis, we further investigated whether macrophage mitochondrial function was impaired during PSCs infection. Flow cytometry analysis of mitochondrial membrane potential and mtROS revealed that PSC infection for 72 h resulted in impaired mitochondrial membrane potential (Figure 3A,C) and elevated mtROS (Figure 3B,D) in macrophages. Measurement of mtDNA copy numbers revealed a progressive decline in macrophage mtDNA during prolonged infection (Figure 3E).

### 3.3. PSCs Infection Causes Macrophage Mitochondrial Network Fragmentation

**Figure 4:**
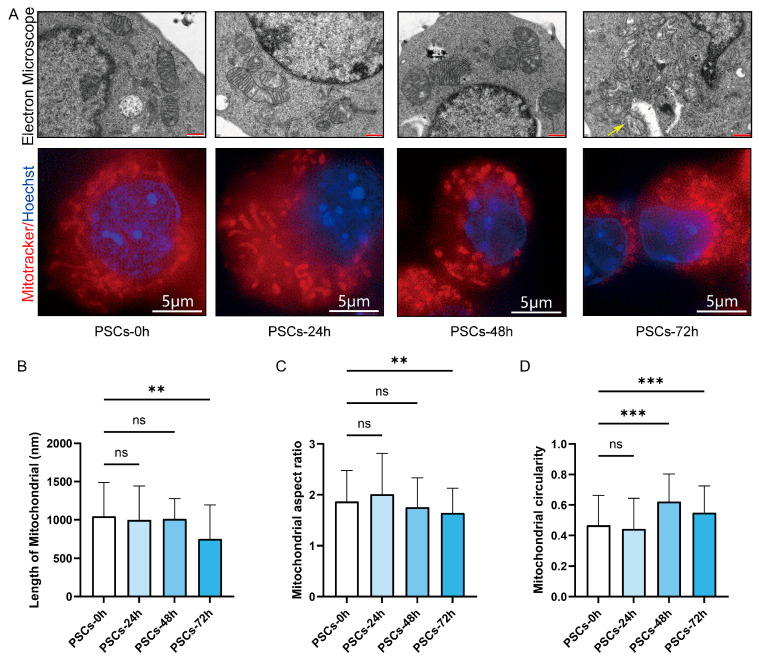
PSCs infection causes Raw264.7 mitochondrial network fragmentation. (**A**) Using TEM and super-resolution confocal microscopy to observe the effects of PSCs infection on the morphology of Raw264.7 mitochondria at 0–72 h. Raw264.7 cells were stained with MitoTracker to detect mitochondria (red) and Hoechst to visualize nuclei (blue). (TEM: red scale bar, 500 nm; super-resolution confocal microscopy: white scale bar, 5 μm). Mitochondrion undergoing the process of mitochondrial fission is indicated by a yellow arrow. (**B**) Statistics of the mitochondrial length of each group in the TEM part of (**A**). The experiments were performed in triplicate; at least 11 cells per group were examined. Statistical analysis was performed using one-way ANOVA, ns, not significant, ** *p* < 0.01. (**C**) Statistics of the aspect ratio of mitochondria in each group in the super-resolution confocal microscopy part of (**A**). The experiments were performed in triplicate; at least three cells per group were examined. Statistical analysis was performed using one-way ANOVA, ns, not significant, ** *p* < 0.01. (**D**) Statistics of the circularity of mitochondria in each group in the super-resolution confocal microscopy part of (**A**). The experiments were performed in triplicate; at least three cells per group were examined. Statistical analysis was performed using one-way ANOVA, ns, not significant, *** *p* < 0.001.

We further examined the morphological alterations in Raw264.7 mitochondria during PSCs infection. TEM revealed that, as infection progressed, mitochondria became progressively shorter and more rounded, accompanied by swelling and loss of cristae definition. Notably, the reduction in mitochondrial length reached statistical significance at 72 h post-infection (Figure 4A,B). Mitochondria were stained with MitoTracker and imaged by super-resolution confocal microscopy. As infection progressed, the mitochondrial network shifted from short rod-like structures to punctate fragments. Quantitative analysis revealed a reduced aspect ratio and elevated circularity, indicating enhanced mitochondrial fission and mitochondrial network fragmentation (Figure 4A,C,D).

### 3.4. The Effects of Inhibiting Macrophage Mitochondrial Fission on Macrophage Mitochondrial Function and Macrophage Function

**Figure 5:**
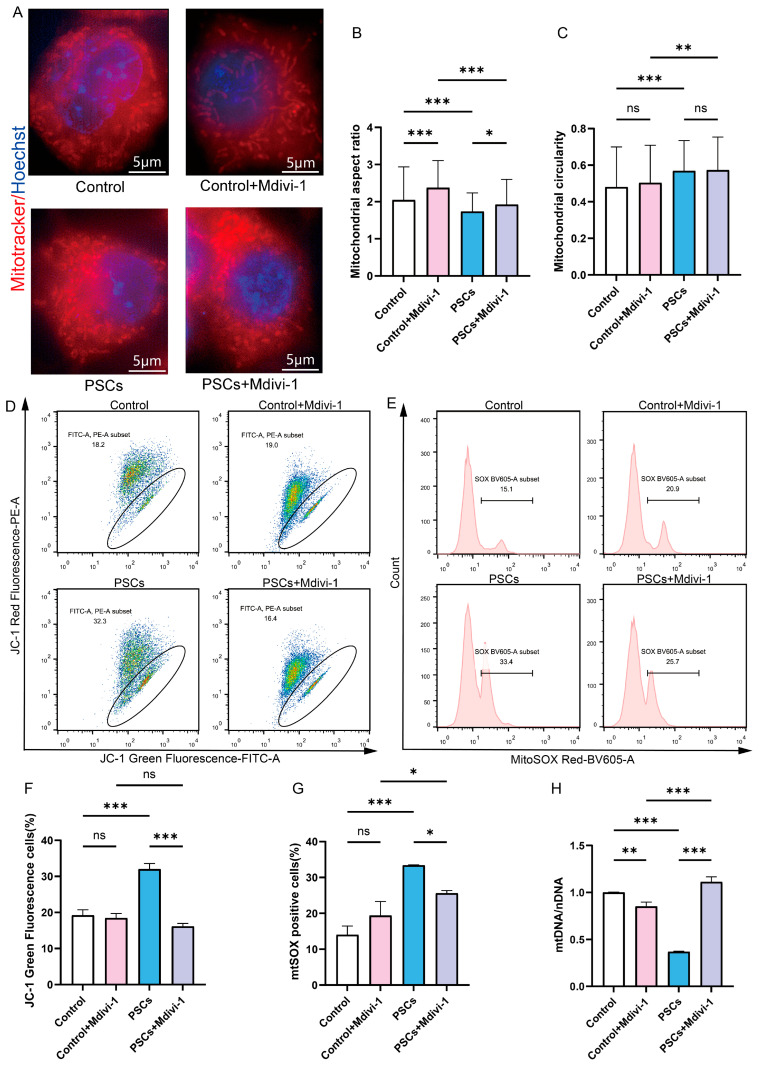
Inhibition of Raw264.7 mitochondrial fission during PSCs infection improves mitochondrial function. (**A**) Super-resolution confocal microscopy was employed to assess mitochondrial morphology in Raw264.7 after a 6 h exposure to 20 μM mdivi-1 (scale bar, 5 μm). (**B**,**C**) Quantification of the mitochondrial morphology in (**A**) (**B**: aspect ratio, **C**: circularity). The experiments were performed in triplicate; at least three cells per group were examined. Statistical analysis was performed using one-way ANOVA, ns, not significant, * *p* < 0.05, ** *p* < 0.01, *** *p* < 0.001. (**D**) The effect of a 6 h exposure to 20 μM Mdivi-1 on the proportion of Raw264.7 with compromised mitochondrial membrane potential was evaluated by JC-1 staining followed by flow cytometry. The JC-1 positive control is illustrated in Figure S1. (**E**) The impact of a 6 h treatment with 20 μM mdivi-1 on the percentage of mtROS-positive Raw264.7 cells was analyzed by flow cytometry. The MitoSOX negative control is illustrated in Figure S1. (**F**) Statistics on the proportion of Raw264.7 cells with damaged mitochondrial membrane potential in (**D**). Data were collected from three independent biological replicates, each comprising three technical replicates. Statistical analysis was performed using one-way ANOVA, ns, not significant, *** *p* < 0.001. (**G**) Statistics on the proportion of mtROS-positive Raw264.7 cells in each group in (**E**). Data were collected from three independent biological replicates, each comprising three technical replicates. Statistical analysis was performed using one-way ANOVA, ns, not significant, * *p* < 0.05, *** *p* < 0.001. (**H**) qPCR was utilized to detect the mtDNA copy number of Raw264.7 cells, normalized to the amount of nuclear DNA. Data were collected from three independent biological replicates, each comprising three technical replicates. Statistical analysis was performed using one-way ANOVA, ** *p* < 0.01, *** *p* < 0.001.

To determine whether mitochondrial network fragmentation in macrophages is associated with mitochondrial dysfunction and macrophage dysfunction during PSCs infection, and whether inhibiting mitochondrial fission can improve mitochondrial and macrophage dysfunction. The inhibition of mitochondrial fission in Raw264.7 cells was achieved through the utilization of Mdivi-1. Super-resolution confocal microscopy revealed that 20 μM Mdivi-1 treatment for 6 h caused Raw264.7 mitochondria to become elongated, with an increase in the aspect ratio of mitochondria (Figure 5A–C). These findings suggest that, under the specified concentration and duration, Mdivi-1 effectively suppresses mitochondrial fission in Raw264.7 macrophages. Mdivi-1 was used during the final 6 h of the 72 h PSCs infection process. Under super-resolution confocal microscopy, it was observed that compared to the PSCs infection group, the PSCs infection group treated with Mdivi-1 had longer mitochondria and a higher mitochondrial aspect ratio, suggesting that mitochondrial fission was inhibited (Figure 5A–C).

**Figure 6:**
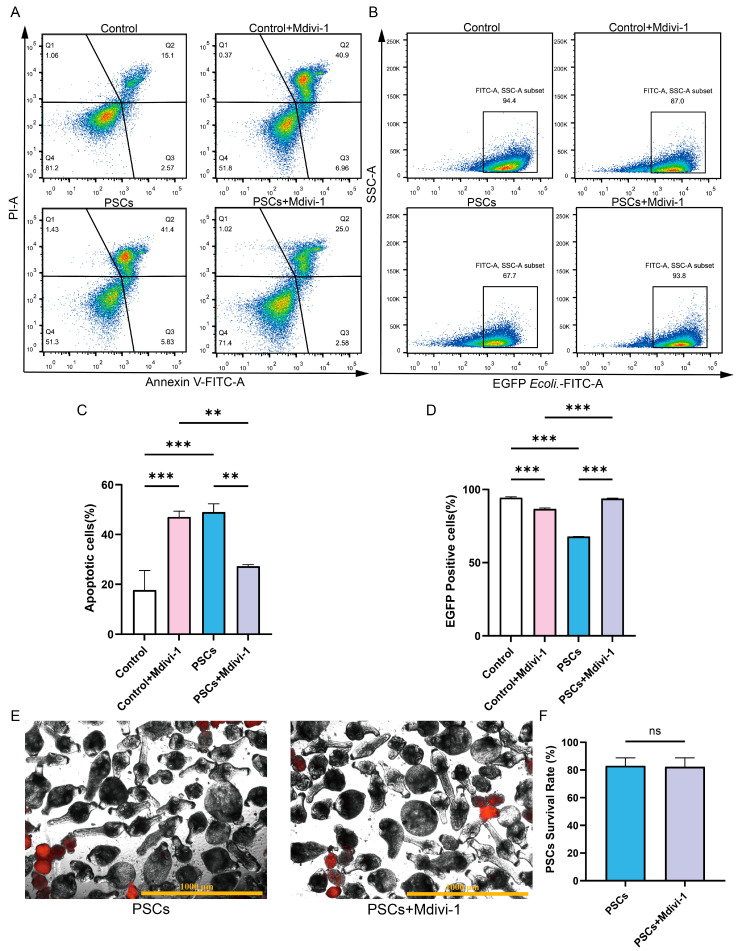
Inhibition of mitochondrial fission during PSCs infection improves Raw264.7 cell function. (**A**) The effect of 20 μM Mdivi-1 treatment for 6 h on the apoptosis rate of Raw264.7 cells was analyzed by flow cytometry. (**B**) The effect of 20 μM Mdivi-1 treatment for 6 h on the proportion of EGFP-positive Raw264.7 cells was analyzed using flow cytometry. (**C**) Statistics of the Raw264.7 apoptosis rate of each group in (**A**). Data were collected from three independent biological replicates, each comprising three technical replicates. Statistical analysis was performed using one-way ANOVA, ** *p* < 0.01, *** *p* < 0.001. (**D**) Statistics on the proportion of EGFP-positive Raw264.7 cells in each group in (**B**). Data were collected from three independent biological replicates, each comprising three technical replicates. Statistical analysis was performed using one-way ANOVA, *** *p* < 0.001. (**E**) Dead PSCs were stained with PI, and the effect of 20 μM Mdivi-1 for 6 h on PSC survival was observed using a fluorescence microscope (Scar bar, 1000 μm). (**F**) The survival rate of PSCs in each group in (**E**). Data were collected from three independent biological replicates, each comprising three technical replicates. Statistical analysis was performed using one-way ANOVA. ns, not significant.

In the absence of PSCs infection, treatment of Raw264.7 with Mdivi-1 for a period of 6 h did not result in alterations to the mitochondrial membrane potential or mtROS levels. However, a reduction in mtDNA copy number was observed (Figure 5D–H). Furthermore, the application of Mdivi-1 to impede mitochondrial fission during PSCs infection has the potential to mitigate damage to the Raw264.7 mitochondrial membrane potential, reduce mtROS levels, and increase mtDNA copy numbers (Figure 5D–H). In the absence of PSCs infection, Mdivi-1 treatment for 6 h promotes apoptosis in Raw264.7 (Figure 6A,C). The administration of Mdivi-1 during the course of PSCs infection has been demonstrated to result in a reduction in apoptosis in Raw264.7 (Figure 6A,C). Phagocytosis constitutes a primary immune function of macrophages. In the absence of PSCs infection, Mdivi-1 inhibited the phagocytic activity of Raw264.7; conversely, during PSCs infection, Mdivi-1 enhanced their phagocytic capacity (Figure 6B,D).

To ascertain whether the improvement in Raw264.7 mitochondrial function and cellular function by Mdivi-1 was due to adverse effects of Mdivi-1 on PSCs, we treated PSCs with Mdivi-1 alone and found that Mdivi-1 did not cause an increase in PSC death (Figure 6E,F).

## 4. Discussion

In this study, the murine macrophage cell line Raw264.7 was infected with PSCs, and it was revealed that infected macrophages exhibited elevated apoptosis and impaired phagocytosis, alongside mitochondrial dysfunction characterized by loss of membrane potential, increased mtROS, decreased mtDNA copy number, and mitochondrial network fragmentation. Inhibition of mitochondrial fission with Mdivi-1 during PSCs infection restored both mitochondrial and cellular functions in Raw264.7 macrophages, indicating that the impaired macrophage activity observed in AE might be attributed to PSCs-induced mitochondrial fission.

Mitochondria are not only organelles that generate energy but also serve as molecular platforms that mediate immune signaling. The present study demonstrates that PSCs induce mitochondrial network fragmentation and impair cellular function in the murine macrophage cell line Raw264.7. Additionally, other parasites promote their survival by inducing fragmentation of the host–cell mitochondrial network; the microsporidian *Encephalitozoon* triggers such fragmentation, and Mdivi-1 treatment markedly curtails *Encephalitozoon* proliferation [31]. Macrophage mitochondrial fission is associated with phagocytic function: our study revealed that PSCs infection elevated mitochondrial fission in Raw264.7 cells and impaired phagocytic activity, whereas inhibiting fission with Mdivi-1 restored phagocytic capacity. Studies on tumor-associated macrophages have shown that mitochondrial dynamics dictate the phase transition of the phagocytic machinery [32]. Concurrently, impaired macrophage phagocytic function is associated with immune suppression occurring in tumors and infectious diseases. Tumor-associated macrophages participate in maintaining the immunosuppressive tumor microenvironment [33]. *Candida albicans* has been observed to suppress the phagocytic function of macrophages. The resultant suppressed macrophages interfere with T cell-mediated immune responses by secreting cytokines, thereby contributing to the formation of an immunosuppressive microenvironment [34]. During *E. multilocularis* infection, the increased number of macrophages surrounding the lesion has been shown to both fail to promote parasite clearance and induce immunosuppression [6]. It was additionally observed that PSCs infection impaired phagocytic activity and increased apoptosis in Raw264.7 cells. Therefore, we hypothesize that *E. multilocularis* may suppress the phagocytic function of macrophages by promoting mitochondrial fission in these cells and inducing their apoptosis. The present study employed the murine macrophage cell line Raw264.7 exclusively at the cellular level to elucidate the relationship between mitochondrial morphology and macrophage function. However, given the established metabolic and immunological differences between cell lines and primary macrophages, further validation in primary macrophages and in vivo experiments in an AE murine model are necessary to ascertain whether the inhibition of mitochondrial fission in macrophages enhances their function and mitigates immune tolerance during the late stages of AE. Additionally, the specific mechanisms by which increased mitochondrial fission in macrophages leads to impaired macrophage function during the course of AE require further elucidation.

## 5. Conclusions

The present study explores the effects of *E. multilocularis* on host macrophages, with a particular focus on mitochondrial fusion and fission. The results indicate that *E. multilocularis* induces increased mitochondrial fission in host macrophages, while impairing both mitochondrial and cellular functions. Inhibiting mitochondrial fission in macrophages during *E. multilocularis* infection has been shown to improve mitochondrial and cellular function. This finding provides insight into the pathogenesis of AE from the perspective of macrophage mitochondrial dynamics. However, further investigation is necessary to evaluate its therapeutic potential for AE.

## Figures and Tables

**Table 1:** Pathogen manipulation of host–cell mitochondrial dynamics favors survival or replication.

Pathogen	Mitochondrial Dynamics Change	Effect on Pathogen
*Legionella pneumophila* [14]	mitochondrial fragmentation	facilitates the replication of *Legionella pneumophila*
*Listeria monocytogenes* [16,17]	transient mitochondrial fragmentation	promote *Listeria monocytogenes* proliferation
Dengue virus	inhibit mitochondrial fission [19]/promote mitochondrial fragmentation [20]	benefit Dengue virus replication
*Leishmania donovani* [22]	increased mitochondrial fission	promote the survival of *Leishmania donovani*
*Chlamydia trachomatis* [23]	mitochondrial elongation and excessive fusion	facilitate the survival of *Chlamydia trachomatis*

## Data Availability

Data supporting the conclusions are included within the article.

## Supplementary Materials

The following supporting information can be downloaded at: https://www.mdpi.com/article/10.3390/pathogens14111097/s1, Figure S1: JC-1 positive control and MitoSOX negative control of Figure 5.

## Abbreviations

The following abbreviations are used in this manuscript:

AE	alveolar echinococcosis
*E. multilocularis*	*Echinococcus multilocularis*
PSCs	protoscoleces
BMDMs	bone marrow-derived macrophages
NLRP3	NOD-like receptor family pyrin domain-containing 3
MAVS	mitochondrial antiviral-signaling protein
mtROS	mitochondrial reactive oxygen species
CCCP	carbonyl cyanide 3-chlorophenylhydrazone
mtDNA	mitochondrial deoxyribonucleic acid
PI	propidium iodide
MFN2	mitofusin-2
MFN1	mitofusin-1
DRP1	dynamin-related protein 1
DENV	dengue virus
TEM	transmission electron microscopy
*E. coli*	*Escherichia coli*
MOI	multiplicity of infection
EGFP	enhanced green fluorescent protein
