# Supplementary figures and images for: Mitochondrial Network Fragmentation Leads to Dysfunction of Macrophages During Echinococcus multilocularis Protoscoleces Infection
Source: Pathogens. 2025 Oct 28;14(11):1097. doi: 10.3390/pathogens14111097 (PMC12654988; doi:10.3390/pathogens14111097)

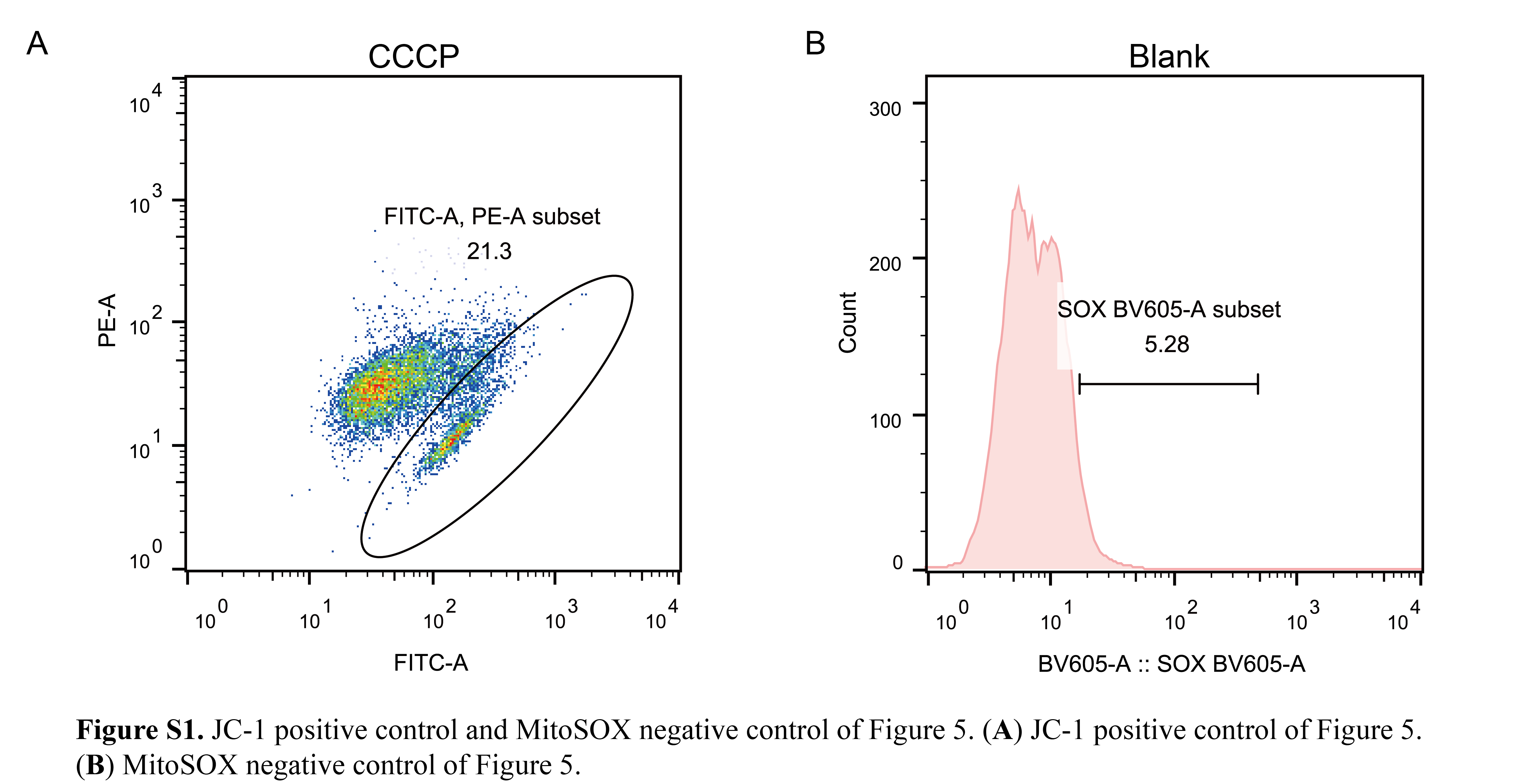

Supplement: Supplementary file 1 [file pathogens-14-01097-s001.zip › pathogens-3884401-supplementary.png]
